# Echocardiographic predictors of outcome in acute heart failure patients in sub-Saharan Africa: insights from THESUS-HF

**DOI:** 10.5830/CVJA-2016-070

**Published:** 2017

**Authors:** Mahmoud U Sani, Beth A Davison, Gad Cotter, Christopher Edwards, Albertino Damasceno, Bongani M Mayosi, Okechukwu S Ogah, Charles Mondo, Anastase Dzudie, Charles Kouam Kouam, Dike B Ojji, Ahmed Suliman, Gerald Yonga, Sergine Abdou, Fikru Maru, Bekele Alemayehu, Karen Sliwa

**Affiliations:** Department of Medicine, Bayero University Kano; Aminu Kano Teaching Hospital, Kano, Nigeria; Momentum Research, Inc, Durham, North Carolina, United States of America; Momentum Research, Inc, Durham, North Carolina, United States of America; Momentum Research, Inc, Durham, North Carolina, United States of America; Faculty of Medicine, Eduardo Mondlane University, Maputo, Mozambique; Department of Medicine, GF Jooste and Groote Schuur Hospitals, University of Cape Town, Cape Town, South Africa; Department of Medicine, University College Hospital, Ibadan and Ministry of Health, Abia State, Nigeria; Uganda Heart Institute, Kampala, Uganda; Department of Internal Medicine, Douala General Hospital and Buea Faculty of Health Sciences, Douala, Cameroon; Department of Internal Medicine, Douala General Hospital and Buea Faculty of Health Sciences, Douala, Cameroon; Department of Medicine, University of Abuja Teaching Hospital, Abuja, Nigeria; Faculty of Medicine, University of Khartoum, Khartoum, Sudan; Department of Medicine, Aga Khan University, Nairobi, Kenya; Department of Cardiology, Faculty of Medecine, Dakar, Senegal; Addis Cardiac Hospital, Addis Ababa, Ethiopia; Addis Cardiac Hospital, Addis Ababa, Ethiopia; Hatter Institute for Cardiovascular Research in Africa, Department of Medicine, Faculty of Health Sciences, University of Cape Town, South Africa

**Keywords:** echocardiography,, acute heart failure,, predictors,, outcome

## Abstract

**Background:**

The role of echocardiography in the risk stratification of acute heart failure (HF) is unknown. Some small studies and retrospective analyses have found little change in echocardiographic variables during admission for acute HF and some echocardiographic parameters were not found to be associated with outcomes. It is unknown which echocardiographic variables will predict outcomes in sub-Saharan African patients admitted with acute HF. Using echocardiograms, this study aimed to determine the predictors of death and re-admissions within 60 days and deaths up to 180 days in patients with acute heart failure.

**Methods:**

Out of the 1 006 patients in the THESUS-HF registry, 954 had had an echocardiogram performed within a few weeks of admission. Echocardiographic measurements were performed according to the American Society of Echocardiography guidelines. We examined the associations between each echocardiographic predictor and outcome using regression models.

**Results:**

Heart rate and left atrial size predicted death within 60 days or re-admission. Heart rate, left ventricular posterior wall thickness in diastole (PWTd), and presence of aortic stenosis were associated with the risk of death within 180 days. PTWd added to clinical variables in predicting 180-day mortality rates.

**Conclusions:**

Echocardiographic variables, especially those of left ventricular size and function, were not found to have additional predictive value in patients admitted for acute HF. Left atrial size, aortic stenosis, heart rate and measures of hypertrophy (LV PWTd) had some predictive value, suggesting the importance of early treatment of hypertension and severe valvular heart disease.

## Background

Recent data clearly indicate that heart failure (HF) is an important healthcare problem in Africa, where it is estimated to constitute about 3–7% of all medical admissions.^1^,^2^ The causes of HF in Africa are different from those outside Africa. The recent THESUS-HF registry^3^ showed that in sub-Saharan Africa, the disease affects men and women in the most productive years of life, at an average age of 52 years. Furthermore, HF in Africa is mostly caused by hypertension and not by coronary artery disease, as is seen in Western countries.^4^

Patients with HF are heterogeneous in terms of risk of cardiac death and re-admission for decompensated HF. Therefore assessment of prognosis is a fundamental step in individual patient management. Analysis of clinical variables has helped in identifying the most significant predictors of mortality in the HF population.^5^

Echocardiography has become the gold standard for the evaluation of patients with HF because it is an inexpensive, highly reproducible, widely available and relatively extensive method for assessing left ventricular systolic and diastolic function.^6^ In fact, the recent HF guidelines of the European Society of Cardiology state that ‘echocardiography is the method of choice in patients with suspected HF for reasons of accuracy, availability (including portability), safety, and cost’.^7^

More than 20 echocardiographic parameters have been proposed as predictors of outcome in HF patients in a number of clinical studies.^8^ However, the role of echocardiography in the assessment and risk stratification of acute HF has been less clear. Some small studies have found little correlation between echocardiographic and haemodynamic variables in acute HF, and little change in these variables from admission to follow up.^9^ In large registries and trials, echocardiographic parameters were not found in many cases to be associated with outcomes.^10^ Therefore, it is not clear which echocardiographic variables are of importance in patients with acute HF.^5^,^11^

THESUS-HF^3^ provided a unique opportunity to study the echocardiographic predictors of outcome in patients admitted with acute HF in this part of the world. To our knowledge, no similar study has been previously published in Africans with acute HF.

## Methods

THESUS-HF was a prospective, multicentre, international observational survey of acute HF in 12 cardiology centres from nine countries in sub-Saharan Africa.^3^ All participating centres had a physician trained in clinical cardiology and echocardiography.

Patients who were older than 12 years, were admitted with dyspnoea as the main complaint, and were diagnosed with acute HF based on symptoms and signs that were confirmed by echocardiography (de novo or decompensation of previously diagnosed HF) were enrolled consecutively. Patients excluded were those with acute coronary syndromes, severe known renal failure (patients undergoing dialysis or with a creatinine level of > 4 mg/dl), nephrotic syndrome, hepatic failure or other cause of hypoalbuminaemia.

Written informed consent was obtained from each subject who was enrolled into the study. Ethical approval was obtained from the ethics review boards of the participating institutions, and the study conformed to the principles outlined in the Declaration of Helsinki.

Details of data collection have been previously described.^3^ In brief, we collected demographic data, detailed medical history, vital signs (blood pressure, heart rate, respiratory rate and temperature) and signs and symptoms of heart failure (oxygen saturation, intensity of oedema and rales, body weight and levels of orthopnoea). Assessments were done at admission and on days 1, 2 and 7 (or at discharge if earlier).

Electrocardiograms were done and read using standard reference ranges. A detailed echocardiographic assessment was performed (see below). The probable primary cause of HF was provided by the investigators, and was based on the European Society of Cardiology guidelines,^7^ as recently applied in the chronic HF cohort of the Heart of Soweto Study.^12^ Information on re-admissions and death, with respective reasons and cause, was collected throughout the six-month follow up. Outcomes of interest were re-admission or death within 60 days, and death up to 180 days.

**Fig. 1.**
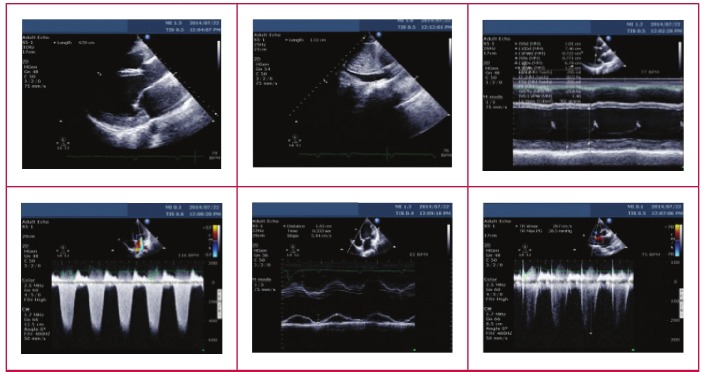
Echocardiography images depicting method of echo assessment in the study.

Echocardiographic procedures and measurements were performed according to the American Society of Echocardiography (ASE) guidelines.^13^ M-mode echocardiograms were derived from two-dimensional (2D) images. The M-mode cursor on the 2D scan was moved to specific areas of the heart to obtain measurements, according to the recommendation of the committee on M-mode standardisation of the ASE. Doppler indices of left ventricular (LV) diastolic filling were obtained. Complete Doppler studies were performed according to the recommendations of the ASE (Fig. 1). From the M-mode measurements, LV dimensions and function (LV ejection fraction) were derived. LV mass was calculated using the recommended method from the ASE:

1.04 [(LVIDd + PWTd + IVSTd)3 – (LVIDd)3] – 13.6 g.^14^

where LVIDd is left ventricular internal diameter at end-diastole, PWTd is posterior wall thickness in diastole, and IVSTd is interventricular septal thickness in diastole.

For diastolic function, left atrial (LA) size (both anteroposterior diameter and planimetry) and pulse-wave mitral-valve (MV) inflow (early and late peak diastolic velocities, which measure the E/A ratio and the deceleration time and MV A-wave duration) were measured.

Echocardiography examinations also included assessment of valvular architecture, a semi-quantitative estimate of the severity of valvular regurgitation, and determination of the presence of pericardial effusion. Other abnormalities, such as evidence of pulmonary arterial hypertension, were also noted.

## Statistical analysis

Patients whose echocardiograph was performed within four weeks prior to and two weeks post enrollment were included in this analysis. Continuous parameters are summarised as means and standard deviation, and categorical parameters by absolute and relative frequencies.

For patients who had their E/A ratios recorded, grade 1 was defined as E/A < 0.8, grade 2 as E/A between 0.8 and 1.5, and grade 3 as E/A ratio > 1.5. If a patient had a missing E/A ratio, then the grade was defined using the E-wave deceleration time as follows: grade 1 as E-wave > 200 ms, grade 2 as E-wave 160–200 ms, and grade 3 as E-wave < 160 ms.

The associations between echo parameters and clinical outcomes were examined using Cox regression models. The univariate associations between each predictor and outcome were examined. The linearity of associations between continuously distributed predictors and each outcome was assessed using restricted cubic splines with four knots with a test of the significance of the non-linear terms. If the association was non-linear, a readily interpretable transformation was chosen through examination of plots of the predicted log hazard ratio against the value of the predictor and changes in Akaike’s information criterion.

For the outcome of 180-day mortality, the associations with creatinine, heart rate and posterior wall thickness were all significantly non-linear. We chose to model creatinine as a quadratic polynomial, and heart rate and posterior wall thickness using linear splines with one knot where the association between predictor and outcome appeared to change.

Univariate associations between echo parameters and outcomes are presented for the whole analysis population as well as by key diagnosis groups. Diagnoses were grouped as hypertension, cardiomyopathy, valvular and other. Valvular was defined as having rheumatic heart disease or at least one of the following classified as severe: aortic stenosis or regurgitation, mitral stenosis or regurgitation. To assess whether an association between an echo parameter and outcomes differed by diagnosis group, we tested for the significance of the diagnosis-by-echo parameter interaction term in the Cox regression model for the outcome.

The number of events in the analysis population limited development of multivariate models for 180- and 60-day mortality or re-admission. Because of this, we chose a few echo parameters in addition to predictors known to be associated with each outcome in this study population.

Multiple imputations were used with a method that assumes multivariate normality (SAS PROC MI) to handle missing values. The imputation model included all covariates under consideration for the multivariate models. The ranges of imputed values were restricted to the ranges of the observed values. Seven imputation datasets were used. Parameter estimates were averaged across these datasets using Rubin’s algorithm (SAS PROC MIANALYZE). Backwards selection was used in each of the seven imputation datasets, with the criterion for staying at p < 0.10. Predictors that were significant in the majority of the imputed datasets were kept in the final model. SAS release 9.2 (SAS Institute, Cary, NC, USA) was used for analyses.

## Results

There was a total of 1 006 patients in the THESUS-HF registry,^3^ of whom 954 had an echocardiogram performed within four weeks before to two weeks after enrollment. Among these 954 patients, the mean age ± SD of the patients was 52.3 ± 18.2 years, 469 (49.2%) were men, the predominant race was black African (99.1%), 11.4% of patients had diabetes mellitus and 9.0% had hyperlipidaemia. The mean left ventricular ejection fraction (LVEF) ± SD was 39.4 ± 16.4%, the initial systolic blood pressure was 130.7 ± 33.5 mmHg, and heart rate was 104 ± 21.4 beats per min (Table 1).

**Table 1:** Patient characteristics overall and by ejection fraction groups

*Patient characteristics*	*Overall (n = 954)*	*EF < 50% (n = 654)*	*EF ≥ 50% (n = 243)*	*p-value*
Age, years, mean ± SD	52.3 ± 18.24	52.3 ± 17.64	53.0 ± 19.58	0.62
Male gender, n (%)	469 (49.2)	342 (52.3)	101 (41.7)	0.0050
Black Africans, n (%)	939 (99.1)	646 (99.1)	242 (99.6)	0.68
Hypertension, n (%)	532 (56.0)	369 (56.7)	138 (57.0)	0.93
Hyperlipidaemia, n (%)	84 (9.0)	58 (9.1)	23 (9.6)	0.80
History of smoking, n (%)	93 (9.8)	64 (9.8)	17 (7.1)	0.20
Malignancy, n (%)	13 (1.4)	10 (1.5)	3 (1.2)	1.00
History of cor pulmonale, n (%)	67 (7.1)	34 (5.2)	30 (12.4)	0.0002
Diabetes mellitus, n (%)	109 (11.4)	72 (11.0)	26 (10.7)	0.88
Peripheral oedema, n (%)	631 (67.1)	448 (69.6)	146 (60.8)	0.014
Rales, n (%)	533 (63.8)	382 (65.3)	130 (59.6)	0.14
BMI, kg/m2, mean ± SD	24.9 ± 5.84	24.8 ± 5.62	24.7 ± 6.10	0.82
Systolic BP, mmHg, mean ± SD	130.7 ± 33.51	127.9 ± 32.16	137.2 ± 36.35	0.0006
Diastolic BP, mmHg, mean ± SD	84.5 ± 21.04	84.0 ± 20.52	85.5 ± 22.04	0.34
Heart rate, bpm, mean ± SD	104.0 ± 21.35	105.0 ± 21.02	101.1 ± 22.69	0.016
LVEF, %, mean ± SD	39.4 ± 16.43	39.4 ± 16.43	60.6 ± 9.65	< 0.001
Creatinine level, mg/dl, mean ± SD	1.4 ± 0.99	1.4 ± 0.99	1.3 ± 1.07	0.54
BUN, mg/dl, mean ± SD	34.7 ± 31.59	35.1 ± 29.58	35.9 ± 38.35	0.79
Sodium level, mEq/l, mean ± SD	135.2 ± 6.57	135.0 ± 6.72	135.5 ± 6.3	0.27
eGFR, ml/min/1.73 m^2^, mean ± SD	84.4 ± 47.91	81.7 ± 44.08	90.8 ± 57.97	0.032
Haemoglobin, g/dl, mean ± SD	12.1 ± 2.41	12.3 ± 2.30	11.8 ± 2.64	0.019
Glucose level, mg/dl, mean ± SD	109.8 ± 49.92	110.4 ± 51.95	106.1 ± 41.93	0.22
(mmol/l)	(6.09 ± 2.77)	(6.13 ± 2.88)	(5.89 ± 2.33)	
Prior medication use, n (%)				
ACE inhibitor	180 (32.4)	134 (34.9)	40 (24.8)	0.022
Loop diuretics	215 (39.4)	152 (40.1)	57 (36.5)	0.44
β-blockers	97 (17.9)	69 (18.3)	26 (16.7)	0.65
Digoxin	103 (18.9)	80 (21.1)	22 (13.9)	0.053
Hydralazine	3 (0.6)	2 (0.5)	1 (0.6)	1.00
Nitrates	10 (1.8)	8 (2.1)	2 (1.3)	0.73
Aldosterone inhibitor	101 (18.5)	77 (20.4)	22 (13.8)	0.075
Statins	27 (5.0)	18 (4.8)	9 (5.7)	0.68
Aspirin	122 (22.2)	91 (24.0)	29 (18.1)	0.13
Anticoagulants	31 (5.7)	22 (5.9)	7 (4.4)	0.49
Aetiology of heart failure, n (%)				
Hypertensive CMP	380 (40.9)	274 (42.5)	86 (37.6)	
Idiopathic dilated CMP	129 (13.9)	120 (18.6)	2 (0.9)	
Rheumatic heart disease	133 (14.3)	75 (11.6)	55 (24.0)	
Ischaemic heart disease	71 (7.6)	57 (8.8)	10 (4.4)	
Peripartum cardiomyopathy	72 (7.8)	59 (9.2)	2 (0.9)	
Pericardial effusion tamponade	45 (4.8)	22 (3.4)	23 (10.0)	
HIV cardiomyopathy	22 (2.4)	12 (1.9)	8 (3.5)	
Endomyocardial fibrosis	11 (1.2)	2 (0.3)	8 (3.5)	
Other	66 (7.1)	24 (3.7)	35 (15.3)	

EF, ejaculation fraction; BMI, body mass index; LVEF, left ventricular ejection fraction; BUN, blood urea nitrogen; ACE, angiotensin converting enzyme; CMP, cardiomyopathy; eGFR, estimated glomerular filtration rate.

Heart failure was most commonly due to hypertension (n = 380; 40.9%), followed by rheumatic valvular heart disease (n = 133; 14.3%), and idiopathic dilated cardiomyopathy (n = 129; 13.9%). Ischaemic heart failure was present in only 71 (7.6%) patients (Table 1).

The distribution and proportion of missing values for each echocardiographic parameter are presented in Table 1. LVEF was available for 897 patients and was missing for 6.0% of patients. LVEF was < 50% in 654 (73%) patients and ≥ 50% in 243 (27%) patients. Patients’ characteristics according to LVEF are presented in Table 1. Patients with HF with reduced ejection fraction had higher proportions of males and peripheral oedema, and lower systolic blood pressure, higher heart rate and lower estimated glomerular filtration rate, on average.

Univariate associations between the echo predictors and the outcomes by diagnosis groups (hypertensive heart disease, valvular heart disease and other) suggest that none of the associations of echo parameters with outcomes differed significantly among the diagnostic groups (Tables 2, 3). Univariate associations of echo predictors with 60-day death or re-admission and with 180-day death are shown in Tables 4 and 5, respectively. Heart rate and left atrial size were associated with death or re-admission within 60 days. Heart rate, left ventricular posterior wall thickness and presence of aortic stenosis were associated with the risk of death up to 180 days.

**Table 2:** Univariate associations between echo predictors and 60-day death or re-admission by diagnosis groups

**	*Hypertensive CMP (n = 338)*		*Valvular (n = 217)*		*Other (n = 399)*		**
**	*Hazard ratio*	**	*Hazard ratio*	**	*Hazard ratio*	**	*Interaction*
*Echocardiographic parameter*	*(95% CI)*	*p-value*	*(95% CI)*	*p-value*	*(95% CI)*	*p-value*	*p-value*
LVEDD (mm)	0.98 (0.95–1.01)	0.15	1.02 (0.99–1.05)	0.29	1.01 (0.99–1.03)	0.49	0.17
LVESD (mm)	0.98 (0.96– 1.00)	0.087	1.01 (0.98–1.04)	0.47	1.00 (0.98–1.02)	0.92	0.20
IVSTd (mm)	0.98 (0.89–1.09)	0.76	0.98 (0.88– 1.10)	0.77	0.93 (0.85–1.02)	0.12	0.64
PWTd (mm)	1.03 (0.91–1.15)	0.68	0.97 (0.85– 1.10)	0.59	0.93 (0.84–1.04)	0.19	0.47
LV mass	1.00 (1.00–1.00)	0.44	1.00 (1.00–1.00)	0.63	1.00 (1.00–1.00)	0.59	0.62
LVEF (%), per 5% increment	1.07 (0.97–1.18)	0.16	0.99 (0.89– 1.11)	0.86	0.99 (0.91–1.08)	0.82	0.42
Left atrial size (A-P) (mm)	1.02 (0.97– 1.06)	0.46	1.01 (0.98– 1.05)	0.57	1.00 (0.97– 1.03)	0.97	0.83
Left atrial size (planimetry) mm^2^	1.00 (1.00–1.00)	0.083	1.00 (1.00–1.00)	0.49	1.00 (1.00–1.00)	0.055	0.73
E/A ratio per doubling	0.93 (0.65–1.31)	0.67	1.67 (0.75– 3.75)	0.21	1.15 (0.85–1.55)	0.37	0.35
E-wave deceleration time (ms)	1.00 (0.99–1.00)	0.65	1.00 (0.99–1.00)	0.24	1.00 (0.99–1.01)	0.73	0.77
MV A-wave duration	1.01 (1.00–1.02)	0.25	1.01 (1.00–1.01)	0.049	0.99 (0.99–1.00)	0.17	0.056
MV E/A ratio grades							
Grade 1: impaired relaxation	(reference group)		(reference group)		(reference group)		
Grade 2: pseudonormal	1.63 (0.66–3.98)	0.32	–		0.78 (0.29–2.09)	0.63	0.18
Grade 3: restrictive filling	0.93 (0.39–2.18)		–		1.13 (0.49–2.58)		

LVEDD, left ventricular end-diastolic diameter; LVESD, left ventricular end-systolic diameter; IVSTd, interventricular septal thickness in diastole; PWTd, posterior wall thickness in diastole; LV, left ventricular; LVEF, left ventricular ejection fraction; A-P, antero-posterior; MV, mitral valve.

Heart rates are for an increment of one unit in the predictor unless otherwise noted. Valvular group defined as rheumatic heart disease or having severe mitral stenosis/regurgitation, aortic stenosis/regurgitation.

**Table 3:** Univariate associations between echo predictors and 180-day death by diagnosis groups

**		*Hypertensive CMP (n = 338)*		*Valvular (n = 217)*		*Other (n = 399)*		**
**	*Hazard ratio*	**	*Hazard ratio*	**	*Hazard ratio*	**	*Interaction*
*Echocardiographic parameter*	*(95% CI)*	*p-value*	*(95% CI)*	*p-value*	*(95% CI)*	*p-value*	*p-value*
LVEDD (mm)	0.98 (0.96–1.01)	0.25	1.01 (0.98–1.04)	0.47	1.02 (1.00–1.04)	0.12	0.17
LVESD (mm)	0.99 (0.96–1.01)	0.28	1.01 (0.99–1.04)	0.32	1.01 (0.99–1.03)	0.19	0.20
IVSTd (mm)	0.95 (0.85–1.06)	0.34	0.99 (0.89–1.09)	0.80	0.91 (0.84–1.00)	0.041	0.50
PWTd (mm)							
≤ 9 mm	0.58 (0.42–0.80)	0.0011	0.79 (0.57–1.10)	0.32	0.82 (0.67–1.00)	0.072	0.30
> 9 mm	1.73 (1.16–2.59)	0.0011	1.38 (0.91– 2.11)	0.32	1.19 (0.88–1.62)	0.072	0.30
LV mass	1.00 (0.99–1.00)	0.097	1.00 (1.00–1.00)	0.85	1.00 (1.00–1.00)	0.62	0.36
LVEF (%), per 5% increment	1.00 (0.90–1.11)	0.99	0.95 (0.85–1.06)	0.36	0.93 (0.86–1.02)	0.11	0.59
Left atrial size (A-P) (mm)	0.99 (0.94–1.03)	0.52	1.01 (0.97–1.04)	0.79	0.99 (0.96–1.02)	0.63	0.79
Left atrial size (planimetry) mm^2^	1.00 (1.00–1.00)	0.70	1.00 (1.00–1.00)	0.78	1.00 (1.00–1.00)	0.34	0.72
E/A ratio, per doubling	1.03 (0.74–1.43)	0.89	2.07 (1.01–4.26)	0.049	1.13 (0.86–1.49)	0.38	0.21
E-wave deceleration time (ms)	1.00 (0.99–1.00)	0.23	1.00 (1.00–1.00)	0.42	1.00 (0.99–1.00)	0.15	0.68
MV A-wave duration	1.00 (0.99–1.01)	0.63	1.01 (1.00–1.01)	0.12	1.00 (0.99–1.01)	0.47	0.26
MV E/A ratio grades							
Grade 1: impaired relaxation	(reference group)		(reference group)		(reference group)		
Grade 2: pseudonormal	1.63 (0.67–3.98)	0.50	0.82 (0.07–8.99)	0.14	2.71 (0.91–8.04)	0.20	0.27
Grade 3: restrictive filling	1.14 (0.50–2.61)		3.01 (0.40–22.68)		2.16 (0.76–6.15)		

LVEDD, left ventricular end-diastolic diameter; LVESD, left ventricular end-systolic diameter; IVSTd, interventricular septal thickness in diastole; PWTd, posterior wall thickness in diastole; LV, left ventricular; LVEF, left ventricular ejection fraction; A-P, antero-posterior; MV, mitral valve.

Heart rates are for an increment of one unit in the predictor unless otherwise noted. Valvular group defined as rheumatic heart disease or having severe mitral stenosis/regurgitation, aortic stenosis/regurgitation.

**Table 4:** Univariate associations between echo predictors and 60-day death/re-admission

*Echocardiographic parameter*	*Hazard ratio (95% CI)*	*p-value*
Heart rate, per increment of 5 bpm	1.07 (1.02–1.13)	0.0088
LVEDD (mm)	1.00 (0.99–1.02)	0.81
LVESD (mm)	1.00 (0.98–1.01)	0.63
IVSTd (mm)	0.96 (0.91–1.01)	0.14
PWTd (mm)	0.97 (0.91–1.03)	0.34
LV mass	1.00 (1.00–1.00)	0.63
LVEF (%), per 5% increment	1.02 (0.96–1.07)	0.58
Left atrial size (A-P) (mm)	1.01 (0.99–1.03)	0.57
Left atrial size (planimetry) mm^2^	1.00 (1.00–1.00)	0.030
E/A ratio, per doubling	1.07 (0.86–1.34)	0.53
E-wave deceleration time (ms)	1.00 (0.99–1.00)	0.13
MV A-wave duration	1.00 (1.00–1.01)	0.43
MV E/A ratio grades		
Grade 1: impaired relaxation	(reference group)	
Grade 2: pseudonormal	1.07 (0.55–2.06)	0.61
Grade 3: restrictive filling	1.28 (0.72–2.26)	
Aortic stenosis		
None, mild	(reference group)	
Moderate	1.83 (0.45–7.41)	0.69
Severe	0.90 (0.22–3.65)	
Aortic regurgitation		
None, mild	(reference group)	
Moderate	1.20 (0.61–2.36)	0.072
Severe	2.42 (1.13–5.19)	
Mitral stenosis		
None, mild	(reference group)	
Moderate	1.56 (0.58–4.22)	0.50
Severe	0.64 (0.20–2.00)	
Mitral regurgitation
None, mild	(reference group)	
Moderate	0.95 (0.63–1.41)	0.95
Severe	1.03 (0.60–1.76)	
Tricuspid regurgitation		
None, mild	(reference group)	
Moderate	1.41 (0.94–2.11)	0.23
Severe	1.21 (0.66–2.21)	

LVEDD, left ventricular end-diastolic diameter; LVESD, left ventricular end-systolic diameter; IVSTd, interventricular septal thickness in diastole; PWTd, posterior wall thickness in diastole; LV, left ventricular; LVEF, left ventricular ejection fraction; A-P, antero-posterior; MV, mitral valve. Heart rates are for an increment of one unit in the predictor unless otherwise noted.

**Table 5:** Univariate associations between echo predictors and 180-day mortality

*Echocardiographic parameter*	*Hazard ratio (95% CI)*	*p-value*
Heart rate		
≤ 80 bpm, per change of 5	0.90 (0.76–1.06)	0.0001
> 80 bpm, per change of 5	1.25 (1.03–1.52)	
LVEDD (mm)	1.01 (0.99–1.02)	0.39
LVESD (mm)	1.01 (0.99–1.02)	0.38
IVSTd (mm)	0.94 (0.89–0.99)	0.025
PWTd (mm)		
≤ 9 mm	0.77 (0.67–0.89)	0.0009
> 9 mm	1.32 (1.08–1.61)	
LV mass	1.00 (1.00–1.00)	0.22
LVEF (%), per 5% increment	0.96 (0.91–1.01)	0.12
Left atrial size (A-P) (mm)	1.00 (0.98–1.01)	0.64
Left atrial size (planimetry) mm^2^	1.00 (1.00–1.00)	0.50
E/A ratio, per doubling	1.13 (0.92–1.39)	0.23
E-wave deceleration time (ms)	1.00 (0.99–1.00)	0.07
MV A-wave duration	1.00 (1.00–1.01)	0.61
MV E/A ratio grades		
Grade 1: impaired relaxation	(reference group)	
Grade 2: pseudonormal	1.77 (0.92–3.38)	0.19
Grade 3: restrictive filling	1.67 (0.92–3.03)	
Aortic stenosis		
None, mild	(reference group)	0.039
Moderate	3.60 (1.33– 9.74)	
Severe	0.83 (0.21–3.36)	
Aortic regurgitation		
None, mild	(reference group)	0.096
Moderate	0.93 (0.46–1.90)	
Severe	2.30 (1.07–4.92)	
Mitral stenosis		
None, mild	(reference group)	0.89
Moderate	0.99 (0.31–3.10)	
Severe	0.79(0.29-2.12)	
Mitral regurgitation		
None, mild	(reference group)	0.87
Moderate	0.92 (0.62–1.34)	
Severe	1.05 (0.63–1.74)	
Tricuspid regurgitation		
None, mild	(reference group)	0.53
Moderate	1.26 (0.85–1.86)	
Severe	1.04 (0.57–1.89)	

LVEDD, left ventricular end-diastolic diameter; LVESD, left ventricular end-systolic diameter; IVSTd, interventricular septal thickness in diastole; PWTd, posterior wall thickness in diastole; LV, left ventricular; LVEF, left ventricular ejection fraction; A-P, antero-posterior; MV, mitral valve. Heart rates are for an increment of one unit in the predictor unless otherwise noted.

The multivariate models suggest left ventricular end-systolic diameter, interventricular septal thickness in diastole, posterior wall thickness in diastole, left atrial size and E/A wave ratio did not add significantly to prediction of 60-day death or re-admission, while left ventricular posterior wall thickness added to clinical variables in the prediction of 180-day mortality (Tables 2, 3).

## Discussion

A thorough and complete echocardiographic examination has been shown to be a useful diagnostic test in the evaluation of patients with HF.^15^ Although it is widely used to evaluate cardiac structure and function in patients with HF, few data are available regarding its ability to predict outcomes.^16^

In the case of acute HF, the precise association of LVEF with cardiovascular outcomes in patients with acute decompensated HF is controversial.^17^ Because the LVEF measure is load dependent and varies with haemodynamic status, it may underestimate or overestimate true myocardial function in various pathophysiological conditions and precipitants of acute decompensation. A prospective study reported that LVEF was weakly correlated with haemodynamic measures and clinical outcomes in patients with acute HF.^18^

Various therapeutic interventions can reduce the risk of re-admissions and death in patients admitted with HF. Therefore, identification of patients at the highest risk of re-admission or death could help provide targeted costeffective interventions. Although several studies have assessed potential echocardiographic predictors, the results have been inconsistent.^19^,^20^ A large number of variables can be measured or calculated by echocardiographic and Doppler imaging. It is not clear which echocardiographic measurements provide independent prognostic information.

In our study, echocardiographic parameters showed only limited associations between echocardiographic measures and outcomes. Heart rate (which can be obtained by simple physical examination) and left atrial size were associated with death or re-admission within 60 days, and left ventricular posterior wall thickness and presence of aortic stenosis were associated with the risk of death up to 180 days. In agreement with the results of the PROTECT study modelling,^10^ LVEF was not associated with 60-day death or re-admission or with 180-day mortality.

This finding contrasts with data from the ESCAPE study, where echocardiographic measures of LV size and function did change from baseline to follow up and were associated with some outcomes.^21^ However, the ESCAPE study enrolled patients with end-stage cardiomyopathy who had very significant LV dysfunction at baseline. These patients were different from the majority of acute HF patients, particularly those enrolled in the THESUS registry.

The results of the current study confirming the preliminary findings of Gandhi et al.^9^ and the retrospective analysis of the PROTECT study^10^ raise the question of why in the general population of patients admitted for acute HF, echocardiographic measures of left ventricular function and size were not associated with outcomes. This puzzling finding suggests that the pathophysiology of acute HF may differ from that of chronic HF by being less dependent on systolic function and, as suggested by Gandhi et al.,^9^ more driven by factors that cause cardiac and vascular stiffening, manifesting as diastolic dysfunction.

Left ventricular hypertrophy (LVH) is a recognised complication of systemic hypertension and the best-studied marker of hypertensive heart disease.^22^ LVH strongly predicts cardiovascular morbidity and mortality in hypertensive patients, and is an independent risk factor for overall cardiovascular mortality and morbidity.^23^ It is known to cause a reduction in myocardial coronary reserve, which predisposes to myocardial ischaemia and left ventricular dysfunction, thereby causing increased incidence of coronary heart disease among hypertensives.^24^ This finding should encourage increased efforts for screening and treatment of young hypertensive patients, in Africa and throughout the world, to prevent the progression of hypertension to LVH.

The increased risk of patients with severe valvular heart disease, particularly aortic stenosis, is well documented.^25^ The confirmation in our study of the increased risk of these patients when admitted for acute HF is important, adding to the evidence encouraging a low threshold for evaluation and treatment of patients with suspected aortic stenosis.

Left atrial enlargement has been increasingly suggested in recent years to be an important indicator of increased risk for an adverse clinical outcome. Left atrial enlargement may serve as an indicator for persistent increased pressure within the cardiovascular system, possibly representing longerterm changes, such as the role of HbA_1c_ in diabetes mellitus. Furthermore, the left atrium modulates left ventricular filling and cardiovascular performance by functioning as a reservoir for pulmonary venous return during ventricular systole, a conduit for pulmonary venous return during early ventricular diastole, and a booster pump that augments ventricular filling during late ventricular diastole. Therefore, left atrial enlargement (and possibly associated dysfunction) may play an important role not only in the marking of cardiovascular dysfunction but also in its enhancement.

The finding that left atrial size is associated with adverse outcomes begs the question of why measures of diastolic dysfunction were not predictive of such adverse outcomes in the current cohort. Although the reasons for that cannot be ascertained, given the limitations of the study (see below), it is possible that, as described by Gandhi et al.,^9^ measures of diastolic dysfunction improve rapidly after admission in patients with acute HF. Because the echocardiographic evaluations performed in the current study were not done close to the time of admission in many patients, the worst measures may have been missed. It is also possible that some specific characteristics of the patient population may have contributed to this lack of association.

Increased resting heart rate is a known predictor for cardiovascular mortality and morbidity in a variety of cardiovascular diseases, including HF.^26^ In patients with reduced LVEF, with or without signs or symptoms of HF, high heart rate has predicted adverse outcomes, irrespective of other known risk factors.^27^ Several pathophysiological mechanisms, including blunting of the force–frequency relationship, the induction of myocardial ischaemia, precipitation of rhythm disturbances, and acceleration of atherosclerosis have been proposed to explain the association between higher heart rate and worse outcomes in patients with HF.^26^

Higher heart rate might also be a marker of greater neurohormonal activation. The SHIFT study showed that heart rate is important in the pathophysiology of HF with reduced LVEF, and that heart rate reduction per se is a mechanism responsible for improvement in clinical outcomes.^28^

The CHARM investigators also found that the value of resting heart rate in predicting worse outcomes was independent of baseline left ventricular systolic function in heart failure.^29^ A higher heart rate was associated with a greater risk of hospital stay for HF, both in patients with reduced and preserved LVEF in a post hoc analysis of the DIG (Digitalis Investigation Group) trial. In predicting mortality, however, higher heart rate was only significant in patients with a reduced LVEF.^30^

Similar to our findings, left atrial size or its surrogates have been shown to predict hospitalisation for HF and death in other studies.^31^ Left atrial size predicts death among high-risk groups, such as patients with dilated cardiomyopathy, LV dysfunction, atrial arrhythmias, acute myocardial infarction, as well as in in the general population.^32^

Left atrial size, aortic stenosis, heart rate and measures of hypertrophy had some value in predicting outcome in our cohort. This may suggest that early diagnosis and treatment of hypertension and valvular heart disease in sub-Saharan Africa should be emphasised to improve outcome.

## Limitations

Our data should be interpreted in the context of their limitations. Unobserved variables may have confounded the results. Not all echocardiographic parameters were available in all patients, limiting the number of parameters for analysis. The variable timing of the echocardiogram and inter-observer variability may have affected the specific results obtained. Furthermore, the number of events was small. Therefore both the variable selection and the parameter estimates for the selected variables are subject to instability.

We also looked at echo predictors of acute HF from various causes. Even though there was no statistically significant interaction between the echo variables, different conditions and outcomes, there could still be a dilutional effect of grouping heterogeneous conditions together.

Finally, our results are drawn from a population of young acute HF patients predominantly with systolic dysfunction. Consequently, these findings may not apply to older patients or to those with preserved LVEF.

## Conclusions

In accordance with previous studies, echocardiographic variables, especially those of left ventricular size and function, were found to have little or no additional predictive value in patients admitted for acute HF. Left atrial size was associated with death or re-admission within 60 days while left ventricular posterior wall thickness and the presence of aortic stenosis were associated with the risk of death within 180 days. There is a need for further studies of echocardiographic evaluation, especially when performed closer to the acute event, to further elucidate the pathophysiology and risk stratification of patients with acute HF.
